# LATERAL ROOT PRIMORDIA 1 of maize acts as a transcriptional activator in auxin signalling downstream of the *Aux/IAA* gene *rootless with undetectable meristem 1*

**DOI:** 10.1093/jxb/erv187

**Published:** 2015-04-23

**Authors:** Yanxiang Zhang, Inga von Behrens, Roman Zimmermann, Yvonne Ludwig, Stefan Hey, Frank Hochholdinger

**Affiliations:** ^1^INRES, Institute of Crop Science and Resource Conservation, Crop Functional Genomics, Friedrich-Ebert-Allee 144, University of Bonn, D-53113 Bonn, Germany; ^2^Center for Molecular Cell and Systems Biology, College of Life Science, Fujian Agriculture & Forestry University, 350002 Fuzhou, China; ^3^ZMBP, Center for Plant Molecular Biology, Department of General Genetics, University of Tuebingen, D-72076 Tuebingen, Germany

**Keywords:** Aux/IAA, crown root, differentiation zone, LATERAL ROOT PRIMORDIA 1 (LRP1), maize, RUM1, transcriptional activator.

## Abstract

Only little is known about target genes of auxin signalling downstream of the Aux/IAA-ARF module. In the present study, it has been demonstrated that maize *lateral root primordia 1* (*lrp1*) encodes a transcriptional activator that is directly regulated by the Aux/IAA protein ROOTLESS WITH UNDETECTABLE MERISTEM 1 (RUM1). Expression of *lrp1* is confined to early root primordia and meristems and is auxin-inducible. Based on its primary protein structure, LRP1 is predicted to be a transcription factor. This notion is supported by exclusive LRP1 localization in the nucleus and its ability to activate downstream gene activity. Based on the observation that *lrp1* transcription is completely repressed in the semi-dominant gain of function mutant *rum1*, it was demonstrated that the *lrp1* promoter is a direct target of RUM1 proteins. Subsequently, promoter activation assays indicated that RUM1 represses the expression of a GFP reporter fused to the native promoter of *lrp1*. Constitutive repression of *lrp1* in *rum1* mutants is a consequence of the stability of mutated rum1 proteins which cannot be degraded by the proteasome and thus constitutively bind to the *lrp1* promoter and repress transcription. Taken together, the repression of the transcriptional activator *lrp1* by direct binding of RUM1 to its promoter, together with specific expression of *lrp1* in root meristems, suggests a function in maize root development via the RUM1-dependent auxin signalling pathway.

## Highlight

Maize *lateral root primordia 1* encodes an auxin-inducible transcriptional activator confined to root primordia that is repressed by direct promoter binding of the Aux/IAA protein ROOTLESS WITH UNDETECTABLE MERISTEM 1.

## Introduction

Plant roots are instrumental for water and nutrient uptake and for the anchorage of plants in the soil (Hochholdinger *et al.*, 2004a). The monocotyledonous model plant *Zea mays* L. exhibits a complex root stock architecture which comprises embryonically formed primary and seminal roots and postembryonic lateral and shoot-borne roots (Hochholdinger and Tuberosa, 2009). The postembryonic root system makes up the major backbone of the plant (Hochholdinger *et al.*, 2004b). Lateral roots are, per definition, roots that emerge from other roots. Lateral roots are initiated in pericycle cells of all root-types of maize and emerge a few days after the formation of the main roots.

In *Arabidopsis*, the initiation of lateral roots starts with the dedifferentiation of pericycle founder cells located at the xylem pole and leads to the formation of a small primordium which finally breaks the outer tissues to emerge from the parental root (Dolan *et al.*, 1993). By contrast, lateral roots in maize are initiated from pericycle and endodermal cells at the phloem poles (Hochholdinger and Zimmermann, 2008).

The plant hormone auxin plays a pivotal role in the co-ordination of almost all developmental processes including root patterning. Auxin maxima in phloem pole pericycle cells are required for the initiation of lateral root primordia in maize (Jansen *et al.*, 2012). The maize *rum1* mutant is defective in the initiation of lateral root primordia in primary roots and displays an 83% reduction in polar auxin transport in mutant primary roots compared with the wild-type (Woll *et al.*, 2005). The *rum1* gene encodes Aux/IAA10 (von Behrens *et al.*, 2011). Aux/IAA (*Aux*in/*I*ndole-3-*A*cetic *A*cid) proteins are involved in auxin signal transduction by interacting with ARF (*A*uxin *R*esponse *F*actor) proteins. These Aux/IAA–ARF complexes regulate the transcription of early auxin-responsive genes such as *Aux/IAAs*, *SAURs*, and *GH3s* by ARF binding to their auxin-responsive elements (AuxREs) in their promoters (Reed, 2001; Woodward and Bartel, 2005). An increase in cellular auxin levels leads to a rapid degradation of Aux/IAA proteins by the 26S proteasome and, as a consequence, ARF mediated transcription of downstream auxin-responsive target genes (Abel, 2007).

The plant specific family of SHORT INTERNODES-RELATED SEQUENCE (SRS) proteins in *Arabidopsis* consists of ten members (Eklund *et al.*, 2010a). The proteins encoded by this gene family display a zinc-finger motif (Fridborg *et al.*, 1999) and two putative nuclear localization signals (NLSs) (Eklund *et al.*, 2010a). Moreover, they contain a C-terminal IGGH domain which was shown to be required for homodimerization (Eklund *et al.*, 2010a). LRP1 (LATERAL ROOT PRIMORDIUM 1), a member of the SRS family, is involved in early lateral root formation in *Arabidopsis* (Smith and Fedoroff, 1995). *Arabidopsis lrp1* transcripts were only detected during the early stages of lateral and adventitious root primordia formation but not at the later stages of primordia development before the emergence of these structures (Smith and Fedoroff, 1995). SWP1, which is involved in the regulation of flower timing, represses *Arabidopsis lrp1* by histone deacetylation (Krichevsky *et al.*, 2009). Yeast two-hybrid assays demonstrated the ability of *Arabidopsis* LRP1 to form homodimers and heterodimers with members of the SRS family (Kuusk *et al.*, 2006).

In the present study it has been demonstrated that *lrp1* activity in maize is regulated by binding of the Aux/IAA protein RUM1 to the *lrp1* promoter. Subsequent analyses demonstrated that LRP1 functions as a transcriptional activator of downstream gene expression which is in line with its localization in the nucleus. In summary, the auxin-inducible LRP1 protein is involved in maize auxin signal transduction downstream of *rum1* which regulates the initiation of lateral and seminal roots.

## Material and methods

### Plant growth and auxin treatment

Seeds of the maize inbred line B73 were germinated in paper rolls as previously described (Ludwig *et al.*, 2013). Five-day-old maize seedlings were treated with 5 μM of the auxin analogue 1-NAA, maize primary roots were subsequently harvested 0, 1, 2, or 3h after 1-NAA treatment (Ludwig *et al.*, 2013), then immediately frozen in liquid nitrogen and stored at -80 °C for subsequent analyses.

### Semi-quantitative RT-PCR and qRT-PCR experiments

Semi-quantitative RT-PCR experiments were performed with RNA extracted from pools of primary root samples. For root-type-speciﬁc expression studies in the wild-type, *lrt1* (Hochholdinger and Feix, 1998) and *rum1* (Woll *et al.*, 2005) mutant background, separate pools of primary roots with a length of >4cm were harvested, frozen in liquid nitrogen, and processed immediately for total RNA isolation using Trizol (Invitrogen). At this stage, lateral root formation was initiated in all wild-type primary roots as illustrated by Feulgen-staining experiments (data not shown). Subsequently, samples were incubated overnight with RNAse-free DNAseI (Invitrogen) and reverse transcription was performed using SuperScriptII (Invitrogen) reverse transcriptase according to the manufacturer’s protocol. PCR was performed using the oligonucleotide primers *ZmLrp1*-semi-fw and *ZmLrp1*-semi-rv (*ZmLrp1*, GRMZM2G077752; see Supplementary Table S1 at *JXB* online) and LA-Taq Polymerase (TaKaRa) following the manufacturer’s guidelines. The housekeeping gene *actin1* (Genebank AC: AY104722) was used as standard with the oligonucleotide primers *Actin*-fw and *Actin*-rv (see Supplementary Table S1 at *JXB* online). All PCR experiments were repeated in four biological replicates to conﬁrm the reproducibility of the results.

For qRT-PCR experiments, total RNA was extracted from pools of different root tissues and subsequently treated with RNase-free DNaseI as previously described (von Behrens *et al.*, 2011). cDNA was synthesized from total RNA via the qScript cDNA SuperMix (Quanta Biosciences, Gaithersburg, MD, USA), and q-RT-PCR experiments were performed as previously described (Zhang *et al.*, 2014). Four biological replicates in three technical replicates for each pool of different root tissues were analysed in qRT-PCR experiments. *Lrp1* transcripts were assayed relative to *myosin* (von Behrens *et al.*, 2011). The oligonucleotide primers *ZmLrp1*-qPCR-fw and *ZmLrp1*-qPCR-rv, and oligonucleocleotide primers *Myosin*-fw and *Myosin*-rv of the reference gene *myosin1* (486090G09.x1) were used to analyse these gene expression patterns (see Supplementary Table S1 at *JXB* online).

Transcript abundance of *lrp1* after auxin induction was assayed relative to *myosin* for each time point. Differential gene expression was determined by Student′s *t* test (*, *P* ≤0.05; **, *P* ≤0.01; ***, *P* ≤0.001; *n*=4).

### Subcellular localization

In order to construct the maize *lrp1*–GFP plasmid, the open reading frame of *lrp1* was PCR amplified from the GAL4DB-LRP1 plasmid (Lab AC: 761) using *ZmLrp1*-*Kpn*I-fw and *ZmLrp1*-*Bsp*HI-rv, and then introduced into the plant transformation vector CF203 at the restriction sites *Kpn*I and *Bsp*HI (Karin Schumacher, University of Heidelberg) yielding a construct containing a constitutive cauliflower mosaic virus (CaMV) 35S promoter at the 5′ end of the coding sequence of *lrp1* and a 3′ in-frame GFP sequence followed by an rbcs E9 terminator (Lab AC: 755). The subcellular localization experiment was performed by transiently transforming the plasmid 35S::*ZmLrp1*–GFP into *Arabidopsis* Col-0 protoplasts grown in suspension culture for 3 d in the dark. Protoplasts were generated according to Negrutiu *et al.* (1987). Transformation was performed with the PEG method (Merkle *et al.*, 1996) and incubated overnight in the dark at 26 °C in MSCol medium (Liu *et al.*, 2003). The transformed protoplasts were directly examined with a HCX PL APO 63x/1.2W CORR water-immersion objective (Leica Microsystems, Wetzlar, Germany) of a TCS SP2 AOBS confocal microscope (Leica Microsystems). GFP was excited with an argon laser at 488nm and the emitted fluorescence was detected with an argon–krypton laser at 509nm. Image processing was performed with Leica Confocal Software (Leica Microsystems). Epifluorescence images were taken from the identical protoplast that was analysed for green fluorescence localization.

### Transient luciferase expression assays

The control effector vector containing the GAL4 DNA binding domain (Lab AC: 1002), the reporter vector with firefly luciferase (LUC, Lab AC: 1004), and the reference reporter vector with Renilla luciferase (Lab AC: 999) were constructed as previously described (Majer *et al.*, 2012). For generating the effector plasmid GAL4DB-LRP1, the full-length coding sequence of *lrp1* was amplified by nested PCR with oligonucleotide primers *ZmLrp1*-luc-fw, *ZmLrp1*-luc-rv and *ZmLrp1*-luc-*Sma*I-fw, *ZmLrp1*-luc-*Kpn*I-rv from cDNA of 5-day-old maize primary roots (see Supplementary Table S1 at *JXB* online). These sequences were subsequently introduced into the control effector at the *Sma*I and *Kpn*I sites (Lab AC: 761). The reporter, effector, and reference plasmids were co-transformed into *Arabidopsis* Col-0 protoplasts (Li *et al.*, 2010b).

LUC assays were performed with the dual-luciferase reporter assay system (Promega) via a TriStar multimode microplate reader LB 941 (Berthold, Bad Wildbad, Germany). Measurement of LUC activity was repeated three times for each transformant and LUC values were normalized with the internal control Renilla LUC values. Each transformation was independently repeated three times.

### *In situ* hybridization analyses

Root samples were ﬁxed in 4% formaldehyde in phosphate-buffered saline overnight, dehydrated in an increasing ethanol series, and embedded in parafﬁn wax (Paraplast plus; Sigma-Aldrich). Tissue sections (7 μm) were prepared using a Leica rotary microtome 2035 and transferred on Superfrost Plus slides (Microm). Templates for all *in situ* hybridization probes were cloned into the pGEM-T Easy vector (Promega) and ampliﬁed using a combination of a gene-speciﬁc and a M13 forward or M13 reverse primer, respectively. All probes were transcribed using an *in vitro* transcription kit containing digoxigenin-labelled UTP (Roche). *In situ* hybridization experiments were performed according to Jackson (1991).

### EMSA (*E*lectrophoretic *M*obility *S*hift *A*ssay)

A 75bp probe containing an AuxRE motif in the central position was amplified from the 2kb promoter sequence of *lrp1* with the oligonucleotide primers *ZmLrp1*-EMSA-fw and *ZmLrp1*-EMSA-rev from *pZmLrp1*-pGEM (Lab AC: 387) and subcloned into pGEM-T easy (Lab AC: 496). EMSA experiments were performed as described in the Promega technical bulletin TB110 (www.promega.com/tbs/tb110/tb110.pdf). The 75bp probe used for the DNA-protein binding reaction was labelled with [γ-^32^P]-ATP (Hartmann Analytic, Braunschweig, Germany) with a T4 polynucleotide kinase (Fermentas, St Leon-Roth, Germany). 30 μg of the RUM1 raw protein extract containing recombinant GST-RUM1 fusion proteins were incubated with [γ-^32^P]-ATP-labelled DNA fragments and 1 μg of poly-(dI-dC) in 10× buffer (100mM TRIS pH 7.5; 500mM NaCl; 10mM EDTA; 10mM DTT) in a total volume of 30 μl. The reaction product was analysed on a 4% non-denaturing polyacrylamide gel. The specificity of GST-RUM1 binding was controlled by using 50× excess of the specific competitor (unlabelled target sequence) and λ-DNA (Fermentas) as the unspecific competitor, respectively.

### Promoter activation assays

The promoter fragments containing AuxRE of *lrp1* were amplified by PCR with oligonucleotide primers *pZmLrp1*-*Xba*I-fw and *pZmLrp1*-*Xho*I-rv (see Supplementary Table S1 at *JXB* online) from *pZmLrp1*-pGEM (Lab AC: 387) using *Pfu* DNA Polymerase (Fermentas) and cloned into the GFP reporter vector (*pZmLrp1*-pGTKan, Lab AC: 747). The full-length open reading frame of *rum1* was cloned into the binary vector pUC-SPYCE which contains a C-terminal c-Myc-tag as previously described (von Behrens *et al.*, 2011). Five micrograms of reporter construct *pZmLrp1*-pGTKan was co-transformed with 20 μg of effector vector RUM1-SPYCE or blank-SPYCE (von Behrens *et al.*, 2011). The pBT8-35SLUCm3 vector was used for normalization of the transformation efficiency (Li *et al.*, 2010a). The transfected protoplasts were incubated as described above. GFP fluorescence was measured after or without treatment with 10 μM MG-132 for 2h. These experiments were replicated independently three times.

## Results

### Phylogeny and synteny of the maize LRP1-LIKE family

The *Arabidopsis SHORT INTERNODES-RELATED SEQUENCE* (*SRS*) gene family comprises ten members. Among those, the *LRP1* gene encodes a protein that is expressed during early lateral primordia formation. To gain a comprehensive overview of this plant-specific gene family of transcription factors in maize, the *Arabidopsis* LRP1 protein sequence (Genbank AC: AT5G12330) was used as a query for homology searches in maizegdb.org. As a result, nine SHORT INTERNODES-RELATED SEQUENCE (SRS) proteins designated LRP1 and LRP1-Like1 (LRL1) to LRP1-Like8 (LRL8) were identified (see Supplementary Table S2 at *JXB* online). All nine maize genes were assigned to the two maize subgenomes which emerged as a consequence of a whole genome duplication 5–12 million years ago. Hence, all members of the maize gene family were already present in the maize genome before the last whole genome duplication (Schnable *et al.*, 2011; www.skraelingmountain.com/datasets/maize_indexed_by_subgenome.csv). Maize *lrl1*, *lrl3*, *lrl4*, *lrl6*, and *lrl8* were assigned to subgenome 1, while *lrp1*, *lrl2*, *lrl5*, and *lrl7* were assigned to subgenome 2. Among the nine genes, three pairs of paralogues were identified (see Supplementary Table S2 at *JXB* online).

**Fig. 1.**
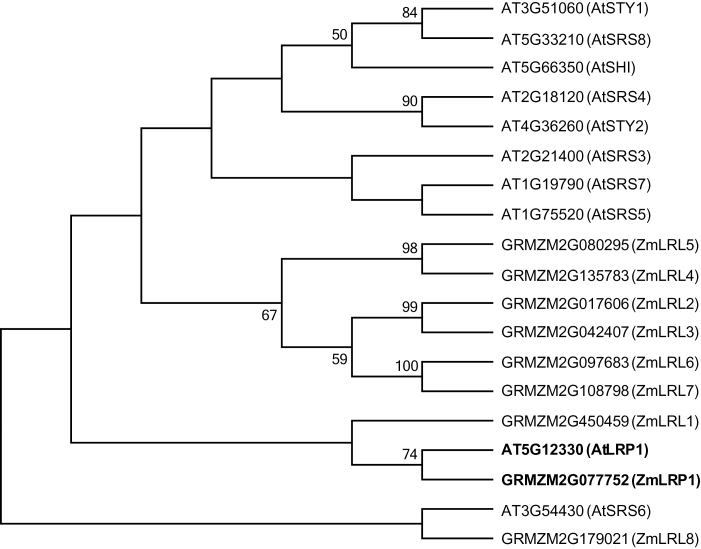
The plant-specific SRS protein family. Phylogenetic relationships of the maize and *Arabidopsis* SRS families were generated by MEGA5. Maize LRP1 and *Arabidopsis* AtLRP1 are highlighted in bold.

To study the relationship between the *Arabidopsis* and maize protein families phylogenetic reconstructions were performed (Fig. 1). The resulting phylogenetic tree revealed that maize GRMZM2G077752, which was designated LRP1, is the closest homologue of AtLRP1. Maize LRL1, which is not a paralogue of maize LRP1, is also closely related to these two proteins. For all other proteins, no one-to-one correlation of homologous pairs was established. The bootstrap values of the two members of a small outgroup AtSRS6 and maize LRL8 were too small to establish a significant correlation between these sequences. All other maize and *Arabidopsis* proteins in Fig. 1 group in an *Arabidopsis*- and a maize specific clade. These phylogenetic relations support the notion of functional diversification of most maize and *Arabidopsis* SRS proteins with the exception of the protein pair maize LRP1/*Arabidopsis* AtLRP1, which might have conserved their molecular function to some extent during evolution.

Based on this result, the maize LRP1 protein was subjected to a detailed functional characterization.

### Maize *lrp1* is expressed in lateral and crown root primordia and is auxin-inducible

**Fig. 2.**
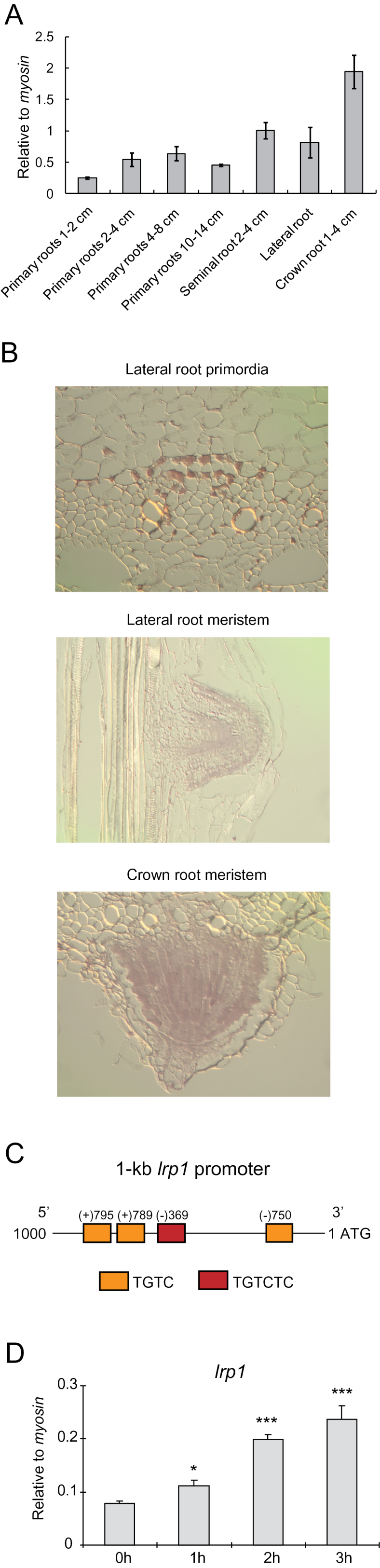
Expression patterns of *lrp1* in root tissues. (A) qRT-PCR analyses demonstrated the expression of *lrp1* in seven specific different root tissues. (B) *In situ* hybridization experiments revealed the expression of *lrp1* in crown root meristems and lateral root primordia and meristems of maize roots. (C) AuxRE analysis of 1-kb *lrp1* promoters of maize. TGTCTC is highlighted by a red box, and TGTC is denoted by a yellow box. (D) Expression of *lrp1* in primary roots of wild-type seedlings after auxin treatment with 5 μM 1-NAA for 0, 1, 2, or 3h, assayed by qRT-PCR relative to *myosin* (*, *P* ≤0.05; **, *P* ≤0.01; ***, *P* ≤0.001; n=4). (This figure is available in colour at *JXB* online.)

Root-type and development-specific expression patterns of maize *lrp1* were analysed in the inbred line B73 by qRT-PCR. Transcripts of *lrp1* were detected in all root types (Fig. 2A). Expression in crown roots was significantly higher than in all other root types under analysis (see Supplementary Table S3 at *JXB* online). By contrast, expression in young primary roots of 1–2cm length was significantly lower than in all other tissues. Finally, expression in seminal roots was significantly higher than in all analysed stages of primary root development but significantly lower than expression in crown roots. In summary, primary, seminal, and crown roots displayed discrete expression levels that distinguished between these root types. Lateral roots displayed a similar expression level as seminal roots that was significantly higher than 1–2cm primary roots but lower than crown roots.

To study expression of *lrp1* in more detail, *in situ* hybridization experiments were performed in postembryonic lateral roots and crown roots. These root types were selected because the formation of these roots can be observed after germination, while primary and seminal roots are formed early during embryo development deep inside the maize seed. In both root types, expression of *lrp1* was confined to early root primordia and meristems (Fig. 2B).

Promoter analysis of 1kb upstream of the ATG start codon of *lrp1* revealed both types of auxin response elements (AuxRE) 5′-TGTCTC-3′ (once) and 5′-TGTC-3′ (three times) (Fig. 2C). Auxin-inducibility of *lrp1* was tested by qRT-PCR in 5-day-old maize B73 primary roots at 5 μM 1-NAA (Fig. 2D). The experiment demonstrated that *lrp1* is auxin inducible with a >3-fold increase of expression within 3h.

### LRP1 is localized in the nucleus and acts as a transcriptional activator

**Fig. 3.**
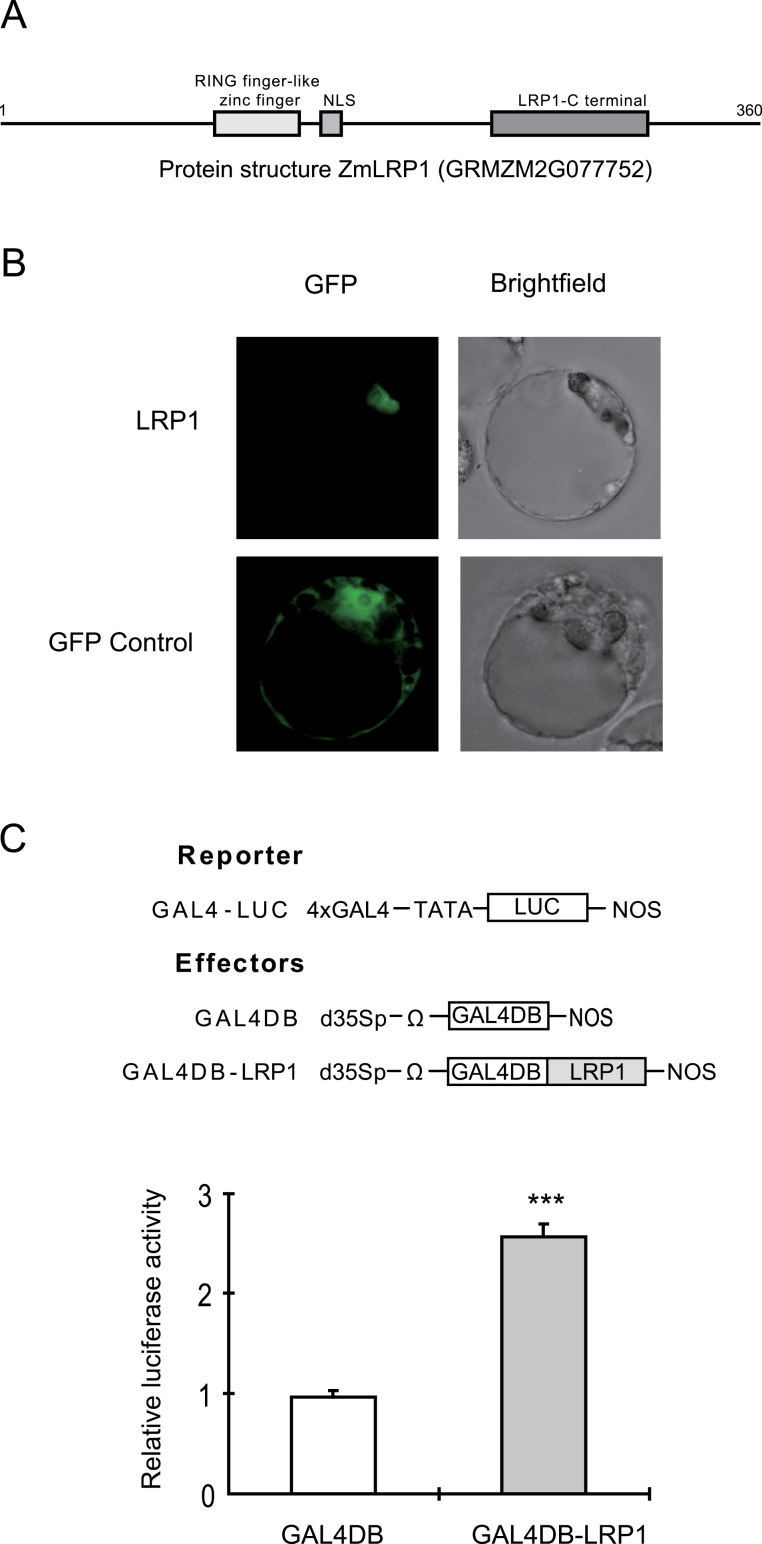
LRP1 acts as a transcriptional activator. (A) The protein structure of maize LRP1. The RING-like zinc finger domain, nuclear localization signal, and LRP1 C-terminal domain are shaded in grey. (B) Subcellular localization of LRP1. The GFP control proteins are localized in both the cytoplasm and the nucleus while the wild-type LRP1–GFP fusion protein localizes only to the nucleus. (C) LRP1 transient co-transfection assay. After co-transformation of *Arabidopsis* Col-0 protoplasts with the reporter construct GAL4-LUC, the effector construct GAL4DB and GAL4DB-LRP1, and the reference construct, the relative luciferase activities are assayed. All luciferase activities are expressed relative to values obtained with the GAL4DB control (GAL4DB set arbitrarily at 1). (This figure is available in colour at *JXB* online.)

The conserved RING finger-like zinc finger, which is a putative DNA binding domain, the nuclear localization signal (NLS), and the LRP1-C terminal domain, which is predicted to be a protein-protein interaction domain (Fig. 3A), suggest that maize LRP1 functions as a transcription factor. To determine the functionality of the predicted nuclear localization signal (NLS) subcellular localization experiments expressing LRP1–GFP fusion proteins in *Arabidopsis* Col-0 protoplasts were performed. LRP1–GFP proteins were exclusively localized in the nucleus (Fig. 3B). By contrast, the GFP control protein displayed a constitutive localization in the nucleus and the cytoplasm (Fig. 3B).

To investigate the capacity of LRP1 to control transcription of downstream genes, a luciferase reporter assay was performed by transiently co-transfecting *Arabidopsis* protoplasts. The effector plasmids consisted of the yeast GAL4 DNA binding domain (GAL4DB) as a control or the GAL4DB fused in-frame with the coding sequence of *lrp1* (GAL4DB-LRP1) driven by a dual 35S promoter (Fig. 3C). The reporter plasmid included the LUC gene driven by the minimal TATA box of the cauliflower mosaic virus 35S promoter with four GAL4-binding sites immediately upstream (Fig. 3C). Co-expression of the GAL4-LUC reporter with GAL4DB-LRP1 effector plasmids resulted in a 2.6-times increase of LUC activity compared with co-expression with the GAL4DB control effector (Fig. 3C). These results suggest that LRP1 activates downstream gene expression and functions as a transcriptional activator.

### RUM1 interacts with the promoter of the *lrp1* gene

**Fig. 4.**
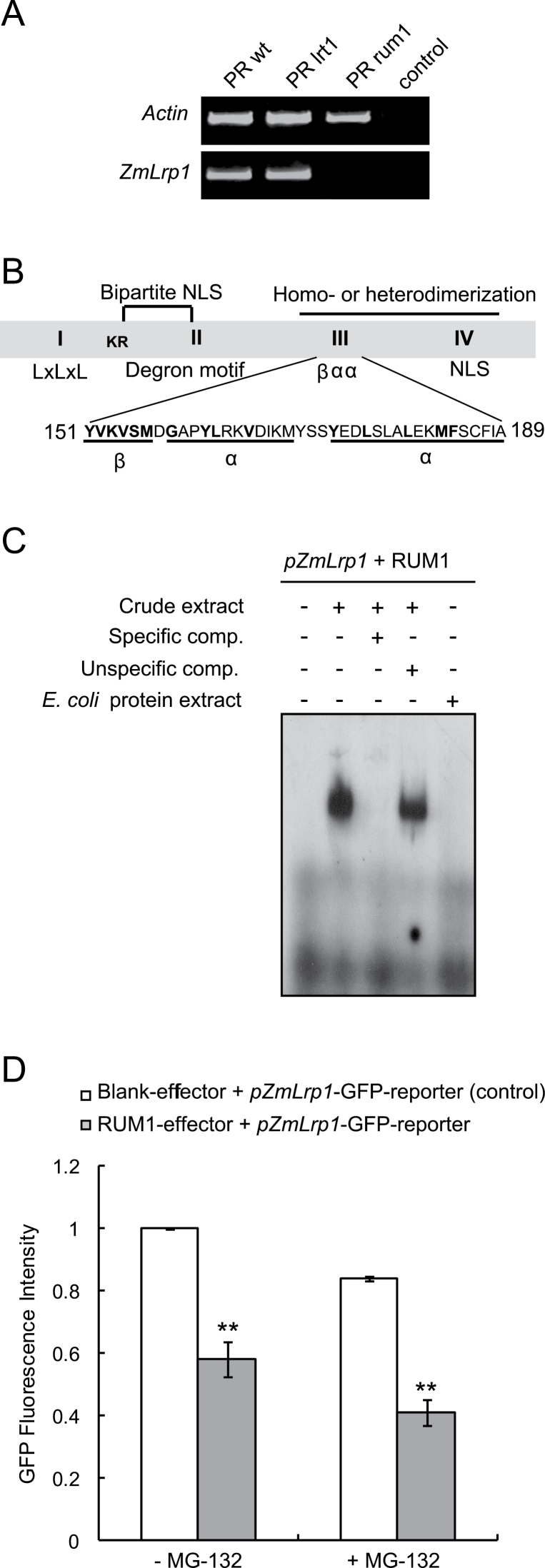
Molecular interaction of RUM1 with *lrp1.* (A) Semi-quantitative RT-PCR with cDNA based on RNA isolated from wild-type, *lrt1* and *rum1* primary roots (PR) indicates an absence of *lrp1* transcripts in the primary root of the mutant *rum1*. The water control was negative. (B) Structure of RUM1 protein with conserved domains I, II, III, and IV. Domain I contains the sequence LxLxL which plays a putative role as transcriptional repressor (Tiwari *et al.*, 2004). Degron motif GWPPV in Domain II is responsible for interaction with the SCF^TIR^ complex. Domains III and IV are involved in homo or heterodimerization (Kim *et al.*, 1997; Ulmasov *et al.*, 1997). Domain III contains a βαα motif which is predicted to act in DNA-binding (Abel *et al.*, 1994). The conserved hydrophobic residues characteristic of the predicted amphipathic βαα fold are in bold. (C) Direct binding of RUM1 to [γ-^32^P]-ATP labelled 75bp *lrp1* promoter fragments containing a central AuxRE motif. A 50× excess of unlabelled 75bp probe was used as a specific competitor while a 50× excess of λ-DNA was used as an unspecific competitor. (D) *lrp1* promoter activation by RUM1. RUM1 effector co-expressed with *lrp1* promoter-driven *GFP* reporter gene (RUM1+*pZmLrp1*–GFP-reporter) in *Arabidopsis* protoplasts under standard conditions or MG-132 treatment after co-transformation. GFP fluorescence intensity was quantified by flow cytometry. The normalization of transformation efficiencies was performed according to Li *et al.* (2010a). The co-transformation of the blank-effector and *pZmLrp1*–GFP-reporter was used as a negative control. Statistical significance between the two experiments were determined by Student′s *t* test (*, *P* ≤0.05; **, *P* ≤0.01).

*In situ* hybridization experiments (Fig. 2B) demonstrated that *lrp1* was expressed in lateral root primordia and meristems. In maize, the mutants *lrt1* (Hochholdinger and Feix, 1998) and *rum1* (Woll *et al.*, 2005) are defective in lateral root formation in primary roots. Therefore, expression of *lrp1* in primary roots of these mutants was measured by semi-quantitative RT-PCR (Fig. 4A). Transcripts of *lrp1* were detected in wild-type and mutant *lrt1* primary roots. However, *lrp1* was not expressed in primary roots of the mutant *rum1* (Fig. 4A) suggesting that this gene is regulated by the Aux/IAA protein RUM1 (AC: GRMZM2G037368) which is a central regulator of auxin signalling. The role of *lrp1* in auxin signalling was supported by the observation that *lrp1* is auxin-inducible (Fig. 2D). Aux/IAA proteins regulate the activity of downstream auxin-responsive genes by protein–protein interaction with ARF proteins. However, it has also been suggested that Aux/IAA proteins might directly bind to the promoter motif AuxRE of auxin-responsive genes (Paciorek and Friml, 2006). RUM1 contains a βαα motif in domain III which was predicted to act in DNA-binding (Abel *et al.*, 1994) (Fig. 4B). Therefore, to test whether *lrp1* is a direct target of RUM1, an EMSA (Electrophoretic Mobility Shift Assay) experiment with recombinant GST-tagged RUM1 was performed (Fig. 4C). It was demonstrated that RUM1 is able to bind to a 75bp promoter sequence of *lrp1* containing an AuxRE (Auxin Responsive Element) motif in a central position (Fig. 4C, lane 2). Specificity of binding was demonstrated by a 50× excess of unlabelled promoter sequence containing the AuxRE motif which prevents sufficient amounts of labelled promoter sequences to bind to RUM1 and generate a detectable shift (lane 3). Moreover, unspecific competition by a 50× excess of λ-DNA which was not bound by RUM1 supported the specificity of the binding (lane 4). To confirm that RUM1 rather than another protein of the bacterial protein lysate binds to the subjected DNA oligonucleotides, crude protein lysate from BL21 cells expressing only GST was applied as a negative control (lane 5).

A promoter activation assay was performed in *Arabidopsis* protoplasts to survey the regulation of *lrp1* gene activity by RUM1. In this experiment, relative GFP fluorescence of a reporter plasmid was monitored. A native *lrp1* promoter fragment was fused upstream of the GFP fragment (*pZmLrp*–GFP reporter) and cotransfected with RUM-effector or Blank-effector constructs (see the Materials and methods). It was demonstrated that the presence of RUM1 repressed the expression of the GFP reporter fused to the native *lrp1* promoter by 42% relative to the control which was arbitrarily set at 1 (Fig. 4D) after transformation efficiency normalization (see the Materials and methods). When the same experiment was performed adding the proteasome inhibitor MG-132, the expression of GFP under the control of the native *lrp1* promoter was further repressed by the presence of RUM1 proteins by 52% relative to the +MG-132 control. This significantly stronger repression in the presence of MG132 is in line with the notion that proteasomal degradation of the Aux/IAA protein RUM1 is inhibited by MG-132, which further represses GFP activity via stabilizing RUM1 binding to the *lrp1* promoter (Fig. 4D).

## Discussion

The *Arabidopsis* SHORT INTERNODES-RELATED SEQUENCE (SRS) family of plant-specific transcription factors comprises ten members (Smith and Fedoroff, 1995). Among those, the *LRP1* gene is expressed during early lateral primordia formation (Smith and Fedoroff, 1995). Homology searches in the maize genome (maizegdb.org) identified a total of nine homologous maize genes designated *lrp1* and *lrp1-like1* (*lrl1*) to *lrl8* (see Supplementary Table S2 at *JXB* online).

About 5–12 mya maize underwent a whole genome duplication which led to the emergence of two subgenomes designated maize 1 and maize 2 (Schnable *et al.*, 2011). Over time, a process called partial fractionation resulted in the loss of one or both copies of the duplicated genes. In the maize *lrp1-like* gene family, three pairs of paralogous genes (66% of the gene family members) have been retained, while for three additional genes (33% of the gene family members) one paralogue was lost by partial fractionation (see Supplementary Table S2 at *JXB* online). Similarly, in the larger *Aux/IAA* gene family of maize, seven pairs of paralogues have been retained (52% of ancient genes) in addition to 13 genes (48% of ancient genes) assigned to a subgenome without a paralogue (Ludwig *et al.*, 2013). Moreover, the maize *Aux/IAA* family also displays seven genes which emerged by single copy duplications after the last whole genome duplication (Ludwig *et al.*, 2013) while no such gene was observed for the *lrp1-like* gene family. In general, partial fractionation resulted in more gene loss in subgenome maize 2 compared with subgenome 1 (Schnable and Freeling, 2011). This tendency was also observed for the *lrp1-like* gene family where two of three (66%) genes assigned to a subgenome without having a paralogue belonged to subgenome 1. Similarly nine of 14 such *Aux/IAA* genes of maize (64%) were assigned to subgenome 1.

Phylogenetic reconstructions revealed that, among the ten *Arabidopsis* and nine maize family members, only for maize LRP1 and *Arabidopsis* AtLRP1 can significant homology based on a bootstrap value >70 be assigned on a one-to-one basis. All other maize and *Arabidopsis* proteins, except AtSRS6 and ZmLRL8 which represent an outgroup, belong to maize- and *Arabidopsis*-specific clades. Hence, for only one of nine (11%) maize *lrp1-like* genes it was possible to determine the *Arabidopsis* homologue (Fig. 1). A similar frequency was observed in a previous phylogenetic study of the LATERAL ORGANS BOUNDARIES DOMAIN (LBD) family of maize (Majer and Hochholdinger, 2011). For only five out of 43 (12%) *lbd* genes, were pairs of homologous unambiguous maize/*Arabidopsis* genes identified (Majer and Hochholdinger, 2011). The difficulty of defining homologous genes between monocot species such as maize and the dicot model *Arabidopsis* is a consequence of the limited colinearity between these species (Brendel *et al.*, 2002).

In the present study, it was demonstrated by *in situ* hybridization experiments that the maize *lrp1* gene is expressed in emerging lateral root primordia and meristems (Fig. 2B). This is consistent with the expression pattern of *AtLRP1* which is involved in *Arabidopsis* lateral root formation (Smith and Fedoroff, 1995). In addition, it was revealed that *lrp1* is also expressed in shoot-borne crown-root primordia of maize (Fig. 2A). The maize root stock is primarily determined by postembryonically formed shoot-borne crown roots while *Arabidopsis* does not form such roots (Hochholdinger and Zimmermann, 2008). Hence, the homologous maize and *Arabidopsis lrp1* genes display conserved expression patterns in lateral roots which are present in both species but also a diversity of expression as a consequence of the structural variation of root stock architecture.

Auxin signal transduction plays a critical role in maize root development as illustrated by root-deficient mutants that are impaired in auxin signal transduction. For instance, the LOB domain protein RTCS controls shoot-borne root initiation (Taramino *et al.*, 2007) while the Aux/IAA protein RUM1 controls lateral root formation in maize (von Behrens *et al.*, 2011). Both genes are auxin-inducible and belong to the classical auxin signalling pathway which also includes the AUXIN RESPONSE FACTORS (ARFs) (Okushima *et al.*, 2007). *Aux/IAA* genes control the activity the auxin signalling mainly by regulating the expression of downstream transcription factors although only few such factors such as LOB domain proteins have been identified (Benjamins and Scheres, 2008; Lau *et al.*, 2011). In the present study it was demonstrated that *lrp1* is a transcription factor involved in auxin signal transduction.

Based on its protein structure containing a DNA binding domain, a nuclear localization signal and a protein–protein interaction domain, maize LRP1 was predicted to be a transcription factor (Fig. 3A). First, it was demonstrated that LRP1–GFP is localized to the nucleus (Fig. 3B). Nuclear localization has also been demonstrated for AtSTY1 another member of this gene family, by transient expression in onion epidermal cells and in protoplasts of the moss *Physcomitrella patens* (Eklund *et al.*, 2010a). Moreover, it was demonstrated that maize LRP1 acts as a transcriptional activator which was confirmed by transient luciferase assays (Fig. 3C). This result was consistent with results for the gene family members AtSTY1 and PpSHI of *Physcomitrella patens* which both act as transcriptional activators regulating downstream transcription (Eklund et al., 2010a, b).

A role of LRP1 in auxin signal transduction based on the observation that *lrp1* transcription is repressed in the semi-dominant mutant *rum1* has been demonstrated in the present study (Fig. 4A). It has been shown that RUM1 binding to the promoter of *lrp1* represses transcription of this gene (Fig. 4C, D). The classical model of Aux/IAA function suggests an indirect control of downstream transcription via the interaction with ARF proteins which bind to downstream auxin-responsive elements AuxRE (Quint and Gray, 2006). Direct DNA-binding of Aux/IAA proteins has been suggested previously (Paciorek and Friml, 2006) although no experimental data for such a function of Aux/IAA proteins were thus far available. In *Arabidopsis*, it was demonstrated that SWP1 represses *AtLRP1* by histone acetylation. The *swp1-1* mutant seedlings showed a consistent increase in primary root length (Krichevsky *et al.*, 2009). AtLRP1 belongs to the family of SHI proteins (Kuusk *et al.*, 2006) which is involved in cell proliferation and expansion in different developmental contexts. Similarly, maize LRP1 could act as regulator of cell division during the early stages of lateral root formation through a RUM1-dependent pathway.

The results obtained in the present study suggest at least two distinct functions for RUM1. It was previously demonstrated that RUM1 acts as a transcriptional repressor interacting with ZmARF25 or ZmARF34, thereby regulating the transcription of auxin-responsive genes in pericycle cells of primary roots (von Behrens *et al.*, 2011). A second function of RUM1 suggested here might be conferred by a direct binding to promoters of downstream genes. The binding of RUM1 to the promoter of *lrp1* has been demonstrated in this study which might play a role in lateral root formation as indicated by the expression pattern of *lrp1* in lateral root primordia. Hence, RUM1 might exert an ARF mediated or direct function as transcriptional repressor regulating the activity of downstream auxin-responsive genes.

## Supplementary data

Supplementary data can be found at *JXB* online.

Supplementary Table S1. Sequences of oligonucleotide primers used in this study (restriction sites underlined).

Supplementary Table S2. Characteristics of the *lrp1-like* gene family in maize (pairs of paralogues are highlighted in different shades of grey).

Supplementary Table S3. Statistical analysis of differential *lrp1* gene expression in various root types according to Fig. 2A.

Supplementary Data
