# Supplementary material for: LATERAL ROOT PRIMORDIA 1 of maize acts as a transcriptional activator in auxin signalling downstream of the Aux/IAA gene rootless with undetectable meristem 1
Source: J Exp Bot. 2015 Apr 23;66(13):3855–63. doi: 10.1093/jxb/erv187 (PMC4473986; doi:10.1093/jxb/erv187)
Supplement: Supplementary Data [file supp_66_13_3855__index.html]

LATERAL ROOT PRIMORDIA 1 of maize acts as a transcriptional activator in auxin signalling downstream of the Aux/IAA gene rootless with undetectable meristem 1 — LATERAL ROOT PRIMORDIA 1 of maize acts as a transcriptional activator in auxin signalling downstream of the Aux/IAA gene rootless with undetectable meristem 1 — Supplementary Data 

# LATERAL ROOT PRIMORDIA 1 of maize acts as a transcriptional activator in auxin signalling downstream of the *Aux/IAA* gene *rootless with undetectable meristem 1*

## Supplementary Data

Data files

**Files in this Data Supplement:**

- Supplementary Data - Supplementary Data
