# Supplementary material for: LATERAL ROOT PRIMORDIA 1 of maize acts as a transcriptional activator in auxin signalling downstream of the Aux/IAA gene rootless with undetectable meristem 1
Source: J Exp Bot. 2015 Apr 23;66(13):3855–63. doi: 10.1093/jxb/erv187 (PMC4473986; doi:10.1093/jxb/erv187)
Supplement: Supplementary Data [file supp_erv187_jexbot145763_file001.pdf]

Supplementary Table S1: Sequences of oligonucleotide primers used in this study (restriction sites underlined).

Myosin-fw	5´ CAAGGAGAGACTCTGTGAGCTTCA 3´
Myosin-rv	5´ AGAAGGCCGTACAGGATCTTACC 3´
ZmLrp1-qPCR-fw	5´ GACATCATCCGCACATTCTC 3´
ZmLrp1-qPCR-rv	5´ ACTCTGCTACTATAGCGTGG 3´
ZmLrp1-semi-fw	5´ TACGTCCTACCCGCCATTTA 3´
ZmLrp1-semi-rv	5´ GCATTGATGCCCTGCTATCT 3´
Actin-fw	5´ ATGTGACAATGGCACTGGAA 3´
Actin-rv	5´ GACCTGACCATCAGGCATCT 3´
ZmLrp1-*Kpn*I-fw	5´ CGGGTACCACCATGGGCCAGGCGGCCGCCATGAACTACGGGATGGCCGACGTG 3´
ZmLrp1-*Bsp*HI-rv	5´ GCTCATGATGTTCATGGTGTTACCGTAGC 3´
ZmLrp1-luc-fw	5´ CCACTCTCTCTCCGCTGCTG 3´
ZmLrp1-luc-rv	5´ GTAGGACGATGGAATGAAGG 3´
ZmLrp1-luc-*Sma*I-fw	5´ TCCCCCGGGACCATGGGCCAGGCGGCCGCCATGAACTACGGGATGGCCGACGTG 3´
ZmLrp1-luc-*Kpn*I-rv	5´ CGGGTACCTCAGTTCATGGTGTTACCGTAGC 3´
ZmLrp1-EMSA-fw	5´ AGAGGAAGCCAGTGATTAGG 3´
ZmLrp1-EMSA-rv	5´ TCTGAGTTGCGCAGCTAGTG 3´
pZmLrp1-*Xba*I-fw	5´ GCTCTAGACTCTCTCTCTTGCAACCTTC 3´
pZmLrp1-*Xho*I-rv	5´ CCGCTCGAGTTGAAGCCGAGGGAGAGGTC 3´

Supplementary Table S2. Characteristics of the *lrp1-like* gene family in maize. Pairs of paralogs are highlighted in different shades of grey.

Name	AC maizeGDB	Maize chromosome	Genome location	Strand	Protein length (aa)	Subgenome	Syntenic paralog
LRP1	GRMZM2G077752_T1	8	157,286,610-157,288,482	1	360	2	
LRL1	GRMZM2G450459_T1	6	143,217,455-143,220,101	1	353	1	
LRL2	GRMZM2G017606_T1	4	216,534,703-216,536,379	-1	296	2	*LRL3*
LRL3	GRMZM2G042407_T1	1	207,433,916-207,443,393	1	302	1	*LRL2*
LRL4	GRMZM2G135783_T2	7	142,938,485-142,940,360	-1	348	1	*LRL5*
LRL5	GRMZM2G080295_T1	2	196,299,773-196,301,206	-1	324	2	*LRL4*
LRL6	GRMZM2G097683_T1	5	60,161,264-60,163,883	1	339	1	*LRL7*
LRL7	GRMZM2G108798_T1	6	85,368,896-85,371,594	-1	331	2	*LRL6*
LRL8	GRMZM2G179021_T1	7	143,145,425-143,146,225	1	221	1	

Supplementary Table S3: Statistical analysis of differential *lrp1* gene expression in various root types according to Figure 2A. Differential gene expression was determined by Student´s *t*-test (*: p ≤ 0.05; **: p ≤ 0.01; ***: p ≤ 0.001).

Primary roots 1 - 2 cm	Primary roots 2 - 4 cm	Primary roots 4 - 8 cm	Primary roots 10 - 14 cm	Seminal root 2 - 4 cm	Lateral root	Crown root 1 - 4 cm	***lrp1***
x	0.02	0.009	0.0002	0.0006	0.03	0.0003	Primary roots 1 - 2 cm
	x	0.28	0.23	0.02	0.17	0.001	Primary roots 2 - 4 cm
		x	0.09	0.04	0.27	0.002	Primary roots 4 - 8 cm
			x	0.003	0.09	0.0006	Primary roots 10 - 14 cm
				x	0.25	0.009	Seminal root 2 - 4 cm
					x	0.009	Lateral root
						x	Crown root 1- 4 cm

	$p \leq 0.05$
	$p \leq 0.01$
	$p \leq 0.001$
